# Extracts

**Published:** 1882-11

**Authors:** 


### Extracts.

---

Infectiveness of Tubercle.—In the course of an address on this subject, by Dr. William Pirrie (*Lancet*) the following specially interesting points occur. It has been said that one-seventh of all the deaths throughout the world and one-third of the deaths in active middle life are caused by tubercular disease. If this be true it must be all important to be familiar with every addition to our knowledge of this disease. The contagiousness of consumption has not been promulgated for the first time in our day, for Galen believed in its contagiousness, and ordered consumptive patients to inhale the vapors from the crater of Etna, while Morgagni, in his day, was of the same opinion. We read also that this belief has always prevailed in Italy, Spain and other countries of Southern Europe. In 1865 Villimin commenced a series of experiments to show that tubercle was an infective disease, and demonstrated the fact that caseation of the tuberculous matter was not necessary to make it a focus of infection. Chauveau, of Lyons, obtained like results from all his experiments, which were of three kinds. First, he gave some oxen, by the stomach, portions of tuberculous matter procured in some instances from man, and in some from oxen, which are very prone, as a class, to what is known by the names of "pearl disease," "angle berries," or "grapes," from the peculiar condition of the serous membrane, or, generally, bovine tuberculosis; second, he inserted particles of tuberculous matter into the connective tissue; and, third, he injected into the veins water into which tuberculous matter had been placed and had been filtered. In all cases tubercle granulations were found in the lungs, and all the animals contracted tuberculosis.

We may here conveniently mention that Dr. Charles Creighton, in his interesting work on bovine tuberculosis in man, published last year, describes numerous cases in which a condition of organs in the human subject was found similar to that seen in oxen dead of tuberculosis; and he establishes the extreme probability that the disease is not unfrequently transmitted to human beings through eating the flesh or drinking the milk of diseased cows. Professor Gerlach also states that healthy oxen can be infected through the milk of sickly cows, or by taking with their food portions of tuberculous matter from the organs of affected animals. Again, in France, M. Giboux operated on rabbits directly with the air itself expired by consumptive patients, with a view to determine whether it could induce tuberculosis in healthy animals. Into one room he placed a cage containing two healthy rabbits, and into it he passed daily, for some time, twenty or twenty-five litres of air expired by phthisical patients. Into another separated room he placed a second cage also containing two healthy rabbits, and into it he passed daily a similar amount of air expired by consumptives, but previously filtered through tow charged with carbolic acid. The consequences were, that at the end of about three months the rabbits in the first-named cage became emaciated and showed signs of disease, and at death tubercles were found in all the organs, but especially in the lungs; whereas, in the second pair of rabbits there seemed to be no injury to the health at the end of a like period, and their organs, when the animals were killed, were found free of tubercles.

We have thus traced a belief in the contagiousness of consumption, held by a few probably in all ages, springing from observation and practical experience; and we have seen how it is justified by the results of experimental research in our own days, and that at last, within the past few months, the precise infecting parasite has been made a subject of microscopic demonstration.

The question now arises, If consumption is an infective disease, can anything be done to destroy its virulence, or to arrest its ravages to a degree hitherto unattained? This much we may safely assert, that the results of all the experiments we have detailed clearly indicate the need of recognizing the parasitic origin of tubercle, of fortifying the body against the inroad, and against all circumstances favorable to the development of the bacilli, as well as of aiming at the destruction of those already pervading the infected organs. With this in view, we must remember the importance of attending carefully to the improvement of the general health of all consumptives; to the avoidance of close confinement in over heated and over crowded rooms, to the keeping the patient as much as possible in the open air in suitable weather; to the careful ventilation of sleeping apartments and sitting-rooms, while guarding against preventable draughts; to the separation of the healthy from the sick as far as possible, at night; and to warning the healthy against all avoidable inhalation of the breath of consumptive patients, who, in their turn, must be kept from re-breathing their own breath. Serious attention should be bestowed also on the disinfection of the sputa of consumptive persons; and much good may be expected also from the more assiduous use of antiseptic inhalations than has been practiced in the past.

Perhaps in all that has been said we may find a plea for cottage hospitals in open, suburban parts, in place of general or large special hospitals, for consumptives; and one, without doubt, for a more careful supervision of the health of cows, whose milk is an article of daily use by the consumptive, and enters so largely into the dietary of all non-consumptives, especially into that of children; as we are told the milk of animals suffering from tubercular dis ease is capable of transmitting it to previously healthy human beings.—*Med. and Surg. Reporter.*

---

Who Should be Medical Students.—The late Professor Rolleston was a strong advocate of the principle of selection applied to candidates for the medical profession, and Sir William Gull at the present time reflects similar opinions in the demand for an "intellectual test" in respect to medical students. Each year new burdens are laid upon these students, both by reason of the ever-increasing ground over which the field of medical study extends, and through the constant additions thereby made to the requirements of examining bodies. There is this peculiarity also about such studies as are included in the medical curriculum, that though they may be easily mastered in the abstract by dint of sheer perseverance, yet it is only a certain class of mind that can successfully appreciate the facts that constitute the main teaching of the wards, a right understanding of which alone enables the student to become an able practitioner. Hitherto the friends of intending students have, as such, paid little attention to any questions of this nature; they have not discussed the likelihood that the destined physician or surgeon may be eminently unfitted to prosecute the labors that will fall to his lot; that his capabilities may be opposed to all chance of successful practice; and that, in his own interest, almost any profession but medicine would have suited him infinitely more advantageously, and with the performance of infinitely better and more serviceable labor to his fellows. It is to this want of perception that we are indebted for the presence in the profession of innumerable unsuccessful men. Often enough they are eminently conscientious, industrious, and well intentioned, but lacking the indispensable qualities that go to make up a skillful practitioner; they plod wearily through life, unrecompensed, and, alas! too frequently unhappy. Unfortunately, also, we cannot even now put into set form of words rules for guidance in deciding on the possession of requisite endowments in any particular case. We recognize that successful practitioners possess certain wellmarked characteristics, and we can easily enough associate their success with such marks of superiority; but it would be difficult to say what traits in a youth will develop into them until we see him at work amidst cases of illness in the wards or out-patient room. Even the most ordinary practitioner requires to possess certain powers of mind in order to be of service to his patients; and it seems but wise that some consideration should be given to the question whether the would-be medical student offers evidence of exhibiting capacity for the profession he is about to enter.

Bodily physique, too, is a detail in this connection not enough regarded. The life led by a medical man is at the best a trying one, and when his lot falls in country districts he must needs be strong in health to overcome all the difficulties of his position. Not a few young men yield early in their career to the strain put upon them by professional work, a strain they have been unable to support; and within our own personal experience cases have occurred in which most promising lives have been early sacrificed in this way. It should, therefore, be always a point for anxious determination whether the incipient student bids fair to grow into a toil supporting practitioner, and mainly because this matter is rarely properly considered we have dwelt upon it in this way at the present time.

As a means of livelihood, in which light it will practically be regarded by a very large number just now, medicine offers very varying prospects. Except as general practitioners, the younger race of qualified men can hope for no immediate return on the capital expended on their education; and it must be at once admitted that the prizes of the profession do not fall to general practitioners. Specialism, howevor much decried, has long been, and will probably long continue to be, the high road to fortune and renown. But only the few can hope for the success represented by a successful consultant s income; the rank and file have to be content with less than this, and it is their fortune to be able to cherish the thought of medicine as the highest, the noblest. and the most deserving of all the arts.—*Med. Press and Circular.*

Hot Baths in Puerperal Convulsions.—Dr. Brieus (*Arch. f. Gynakol.*), reports the following cases:

(1). A primipara, aged thirty-four, after having had sixteen convulsions in one day, was brought to the clinic. During one of the convulsions, she threw herself from the bed and dislocated her right shoulder. After the convulsions she was totally unconscious. Œdema was general, but especially marked on the inferior extremities and the face. The urine contained a large amount of albumen. Pregnancy had advanced to the ninth month. The fœtus was still alive. Within five hours after admission to the hospital and after four more convulsions, two grams of chloral were given by the rectum. On the next day the patient was unconscious, but had no convulsions. The dislocation was reduced. Some days later consciousness returned, and the convulsions reappeared. On the whole, her condition was unchanged. On the fourth day the patient was given a hot bath and then surrounded with warm packing. There was abundant perspiration, followed by sleep, and the œdema and albuminuria rapidly disappeared. A hot bath was given daily, and after the fourth the œdema had wholly disappeared and the amount of albumen in the urine had greatly diminished. After ten days the patient was dismissed from the hospital. There was no return of the convulsions, and after four weeks the woman was delivered of a healthy child.

(2). A lady, thirty-one years of age, in the sixth month of pregnancy, showed marked œdema of the face and the whole body, especially of the genitals. The urine was small in amount and highly albuminous. On the second day after admission she had convulsions, followed by loss of consciousness. She was given three grams of chloral by the rectum and a hot bath (35° Reaumur) of half-hour duration, then surrounded by warm packing. On the following days the baths were repeated. The œdema rapidly subsided and wholly disappeared after the fourth day. The urine became less albuminous, and after six weeks the woman was delivered of a healthy child.

(3). A primipara, aged nineteen, at the time of delivery had marked œdema, albuminuria and two convulsions, followed by stupor. Three grams of chloral were given. After four hours there was another convulsion. Birth was effected by instruments. In an hour there was another convulsion. Two grams of chloral were now given and the woman placed in a hot bath, followed by warm packing. There was no further accident. Four baths in all were given. The œdema and albuminuria disappeared, and the puerperal period passed without disturbance.

(4). A primipara, twenty seven years of age, with marked œdema and albuminuria, had a violent convulsion as the head was born. Two grams of chloral were given without effect. There was a second convulsion, when the hot bath was used. Afterwards there was no accident. On the next day two baths were given. The œdema disappeared, and on the ninth day the urine was free from albumen. There was slight puerperal endometris and tardy involution of the uterus.

(5). A primipara, aged twenty-seven, was brought to the hospital in a deep stupor after repeated convulsions. Delivery was effected under narcosis. Then a hot bath was given, but a paroxysm followed. The patient remained in a stupor, and despite hot baths and chloral, died the next day. Section showed Bright's disease in the second stage, with the kidneys doubled in size. There was marked œdema of the brain and slight of the lungs. There was some cirrhosis of the liver. The uterus had contracted normally.

(6). A primipara, aged twenty, had a delivery without accident, October 11. Afterwards parametritis developed, and November 1 she complained of pain in the eyes and head. The lower extremities became œdematous and amaurosis appeared. The urine was highly albuminous. On November 3 she had a convulsion. She was now given a hot bath, followed by warm packing. The baths were repeated, and by November 28 she had wholly recovered. —*Translated by Ralph D'Ary, M.D., in Physician and Surgeon.*

---

Rational Treatment of Menorrhagia.—Of all the organs in the body, the uterus is the only one from which blood flows as a normal physiological process, the function being influenced by many and various causes, both general and local.

A recognition of this fact is essential; for, unless we acknowledge the importance of forming a correct diagnosis in every case of hæmorrhage, our treatment will not only often be futile, but actually mischievous. Diagnosis is, in fact, the most important element of treatment, for menorrhagia is merely a symptom, not a disease. In many instances, the differentiation of the predisposing and exciting causes of any individual case under observation may be one of great difficulty, but must nevertheless be attempted. The history of the onset of the attack, whether gradual or sudden, attended or not by pain or febrile disturbance, will often give us some clue to the cause. The age of the patient often suggests the possibility of certain well defined causes, cardiac complications from rheumatic fever, hæmatocele, ovarian irritation, constipation, etc., in the young; polypi, fibroids, retroflexion, retained products of conception in the middle-aged; climacteric irregularities, cancer in its various forms, hepatic disorders, between the ages of forty and fifty.

In young plethoric girls, whose sexual development is well marked, menstruation is not unfrequently profuse. In place of giving iron, which generally produces constipation, and thus aggravates the tendency to menorrhagia, the better plan is to regulate carefully the diet, avoiding alcohol and any undue amount of animal food; to give bromides, which lessen the ovarian irritation, together with some saline aperient when requisite.

In anæmic patients, iron generally proves most serviceable; but, in place of "pouring in iron," as is not unfrequently spoken of, it should be given in combination with salines in moderate doses, more as a chalybeate water than a mixture; and care must be exercised that it does not produce headache, nor spoil what little appetite may exist.

In single patients where menorrhagia is marked, and relief does not follow ordinary medical treatment, local investigation should be suggested, and, if necessary, insisted on.

Where a married patient suffers from menorrhagia, the cause of which is not at all obvious at first, a careful examination should be insisted upon. In no fewer than four instances recently, I have met with cases of unsuspected pregnancy complicated by polypus uteri.

N. S., aged 36, married nine years, sterile, began to have irregular losses of blood per vaginam, in addition to the periods being profuse. Ergot was given freely, but no examination was made. When I first saw her, she was looking very anæmic, from a rather smart attack of hæmorrhage. On examining her, I found that the uterus was enlarged above the umbilicus, the cervix soft and full; it was evidently a case of utero-gestation. Protruding from the cervix was a polypus of the size of a walnut. Torsion was employed, and the growth removed, pregnancy advancing to full term.

Where the slightest irregularity in the appearance of the catamenia leads to the suggestion of the possibility of utero-gestation being present, any attack of menorrhagia, especially if it recur, should be regarded as a threatened miscarriage, and treated accordingly. Should only the fœtal portion of the ovum be expelled, hæmorrhage recurring so soon as the patient leaves the recumbent position, in place of contenting ourselves with giving ergot, an examination should be made, and the remainder of the ovum extracted. Where the cervix has closed up so much as to prevent the expulsion of the placenta, though this is rare, a sponge-tent should be inserted, the cervix dilated, and means taken to clear out any debris remaining in the uterus.

Where uterine hæmorrhage is severe, whether from imperfect expulsion of an early ovum, intra-uterine polypus, submucous fibroid tumor, or other similar condition, in place of attempting to restrain the flow by pieces of linen or cotton-wool packed in the vagina, which practically have no influence in controlling the loss in the majority of cases, a far more scientific and rational method is to insert a sponge-tent into the cervix uteri. This has the double effect of plugging the os uteri, thus checking or arresting the flow, and at the same time dilating the cervix, so as to facilitate subsequent exploration of the interior of the uterus.

Where the practitioner does not possess the requisite skill, or has not the instruments at hand, packing the vagina with pieces of sponge is far more likely to arrest the hæmorrhage than pledgets of cotton-wool, which, in place of expanding like sponge when moistened, become compressed and lessened in bulk.

In place of applying hot fomentations to the abdomen, and pouring in brandy, a more rational method of attempting to restrain hæmorrhage is to get the patient rapidly under the influence of opium, so as to allay pain and prevent restlessness, and apply cold or pressure to the abdomen by means of ice or small pads of cotton-wool and a firm binder.—*Arthur W. Edis, M.D., F.R.C.P., in British Med. Jour.—Med. Gazette.*

---

The Hygiene of Trades.—The Hygiene of Trades is such a very extensive subject, that I cannot do more, in a a single lecture, than give you the barest outline. I am sorry to say, and as a native of Great Britain I say it with shame, that although the labor of our artizans has, during the present century, been the chief means of raising the country to its pre-eminence of influence and power, no treatise in the English language has been produced on the Hygiene of Trades, with the exception of a short work, now antiquated, written by Mr. Thakrah, of Leeds, in 1832; whilst in France, Italy, and Germany, numerous treatises have appeared.

The classification of the various occupations, for the purposes of a medico scientific investigation, is a matter of much dificulty, for almost every occupation exposes those engaged in it to more than one source of danger; but they may be divided into three great groups as follows:—

1st. Occupations involving the introduction of deleterious matters into the body.

2nd. Occupations involving exposure to conditions that interfere with nutrition.

3d. Occupations involving exposure to mechanical violence.

In these groups there are comprised over 150 different occupations; but I will confine my observations to one disease, and one class of occupations; and I shall select one disease of very frequent occurrence—viz, phthisis or consumption, and consider it in its relative frequency amongst workmen working in *dusty* trades.

Of tradesmen exposed to *metallic* dust, needle-makers and file-makers head the list, as to susceptibility to consumption, and these occupations are among the most perilous that can be followed. In grinding operations, the various steel and iron instruments are pressed upon grindstones revolving at the rate of 2,000 or 3,000 times a minute; and, in the operation, considerable physical strength is required, and a very constrained position of the body is necessary. The danger of the operation reaches its maximum in the grinding of forks and needless, which must be done on dry stones to avoid the possibility of rust. Razors, scissors, table knives, etc., are ground first on dry stones, and then on wet ones, and danger to the workmen is proportionately diminished as wet stones are used.

The inhalation of *mineral* dust gives rise to the ordinary symptoms of pulmonary consumption, and the lungs are found to contain numerous nodules, varying in color from light gray to deep black, with generally a bright whitish nucleus which have been found, on chemical analysis, to consist of accumulations of mineral dust. The most deadly of all the occupations of this class is that of the flint cutter, the cases of consumption being in the ratio of 80 per cent. Those engaged in the manufacture of Portland cement are exposed to clouds of dust while shoveling the mass into sacks after it has been burned and ground. They are generally troubled with a persistent cough, and they expectorate little lumps of cement. Those thus engaged find it impossible to continue their work day after day, and are obliged to take intervals of rest.

The manufacture of glass is a considerable industry in this city, and I shall, therefore, notice it a little more fully. The workmen who grind and powder the silicious material inhale great quantities of very irritating dust, and suffer much from a hacking cough and inflammation of the eyes. It is said to be rare to find a sound man among them, and they are not able to continue long at work.

Mineral and metallic dust are by far the most active in producing disease, and with regard to both descriptions of dust it has been very clearly established that the finer and drier the dust the more injurious are its effects, as it passes deeper into the lungs, and can only be expectorated with difficulty, or not at all.

Workmen who inhale *vegetable* dust do not suffer very greatly in consequence—unless in cases where great carelessness prevails. Cabinetmakers and carpenters do not suffer much from this cause; but the dust of the hard woods—as mahogany, rosewood, etc.—does cause irritation of the larynx and trachea, and a tendency to bronchial catarrh.

Chimney-sweeps were formerly subject to a peculiar form of epithelioma or cancer, called "chimney-sweeps' cancer"; but the disease is now rarely seen.

Millers suffer much more than bakers from the inhalation of the dust of flour, but it seems to have but little effect upon the respiratory tract, though there is a form of disease called "millers' asthma."

Of all the vegetable dusts, cotton fibre appears to be the most injurious, causing great irritation of the windpipe, frequent cough, and expectoration of a sputa containing cotton fibres. The mortality is found to be greatest among women. The mortality amongst cotton operatives was much greater in former times than now; and a form of disease, called "cotton consumption," was of frequent occurrence, especially in continental factories. The heavy rate of mortality from consumption and other diseases of the lungs, among operatives in textile works, furnished the grounds for an official enquiry by the Medical Officer of the Privy Council. Dr. Greenhow's reports were made to the Privy Council in 1858-60-61, and it was shown by him that in mills, whether flax, cotton, or silk, where the article was manufactured, it was in the preparatory processes that most dust was given off, and most injury to the health of the operatives was caused. These processes are those of sorting, carding, dressing, combing, and hackling. The carding machines of cotton mills were prolific of dust from the carding process, and also from that of preparing, grinding, and cleaning the machine from day to day, or even several times a day. The atmosphere of the spinning rooms has to be kept at a high temperature—about 84° Fah.; and in some mills where the finest thread is made. the temperature is kept as high as 104° Fah., so that the operatives are exposed to great variations of temperature, giving rise to chest affections. Modern ingenuity, however, has devised a plan whereby the carding machine can be made self-cleansing, and otherwise to set itself in working order without manual interference, so that the evolution of dust has been reduced to a minimum in these processes. Similar improvements have been effected in the machinery for weaving silk and woollen goods, and these occupations are now rendered comparatively innocuous.

The manufacture of linen is a very unhealthy occupation. The heating and hackling of flax and hemp cause the evolution of a great deal of dust; and flax hackling in particular is one of the most unhealthy of trades.—*James Christie, A.M., M.D., in Sanitary Journal, Glasgow.*

---

The Continuous Inhalation of the Vapor of Slacking Lime in the Treatment of Membranous Laryngitis. —I procured two narrow pieces of board and fastened them in an uprigbt position to the foot-posts of a double bed. Their tops were connected with each other, and also with the end of the head-board of the bed, by means of rope; a piece of rope also extended from the centre of the head-board to the lower cross rope. Over this rope-work arrangement I placed sheets, pinning them securely and forming a sort of tent about four feet in height and breadth and six feet in length, and covered on all sides. The child and its mother (who was willing to submit to anything that promised to save the life of her little darling) were now placed in this tent and a vessel of water soon set to boiling within it. Into this boiling water every few minutes a lump of lime was thrown, and this was kept up faithfully day and night for five days. To secure a still greater effect, two basins of water were also placed inside the tent, in which lime was slacked from time to time. During this time the mother remained with the child, feeling, like myself, assured that safety depended upon the faithful execution of my orders. She did not, however, find it oppressive, although both herself and the little patient were constantly bathed in perspiration. She even recovered from an unusually severe cold from which she was suffering at the beginning of the treatment. The fact that no great discomfort was experienced in so confined an atmosphere must be ascribed to the absorption of the carbonic acid of the expired breath by the lime, the odor of which was very perceptible in the atmosphere of the tent.

Dr. H. F. Hill, of Baltimore, has proposed and employed with satisfaction a modification of this method. He placed the child on a mattress on the floor beneath a table, over which a sheet was thrown. But this could only be done with older children; to carry out this method effectually, the presence of the mother or nurse within the tent is generally essential.

Of the desirability of the *continuous* use of an agent, the solvent effects of which upon diphtheritic and croupous membranes is capable of such ready and positive proof, in a disease of such terrible fatality as membranous laryngitis, would seem to need no argument. The effect under any circumstances must fall far short of that witnessed in the artificial experiment, where the agent comes in contact with the membrane on all sides. Time, then, is an all-important element in the treatment, and its value is impressed upon us by all writers. Yet, so far as my knowledge extends, no one has sought to obtain the continuous effect of lime, or has suggested a method for securing it readily available.

In conclusion, the advantages of the method proposed may be stated to be—

1. It is simple, safe, economical and universally applicable.

2. It secures the most efficient form in which the application of lime can be made, and is that most nearly approaching the artificial solution outside the body.

3. It secures the *continuous* use of an agent, generally recommended intermittently, and whose utility is unquestionable, and it is the only method that will do this.

4. It secures at the same time, and without extra trouble, the advantages of an atmosphere saturated with steam.—*Eugene F. Cordell, M.D., in Maryland Med. Journal.*

---

Hypertrophied Tonsils.—It frequently happens that the palatine tonsils, which are enlarged and subject to inflammations with abscesses, require special attention. Such often keep up a chronic inflammation of the pharynx just as a foreign body would, producing mechanical irritation and disturbances of circulation; they also interfere with the movements of the palate, and, by pressing its posterior surface upwards against the tubal orifices, narrow or even close those passages. In children and young persons, the removal of hypertrophied tonsils improves the condition of the ears and of the hearing often in a marked degree, and is always favorable to the chronic pharyngeal and nasal catarrh. After the operation, the subacute attacks, with earache and increased deafness, which have previously been very frequent, either cease entirely or are much less common, and the pharyngeal and nasal cavities yield much more readily to the local therapeutics which before the operation were useless. In the children of families where there is an hereditary tendency to catarrhal deafness, enlarged tonsils should be early removed as a prophylactic measure, even where the ears are intact. It is well known that the extirpation of hypertrophied tonsils has a favorable influence on the articulation and voice, and also upon the position of the mouth and the facial expression, that after the operation children breathe much deeper, and consequently that the chest becomes more fully developed, the blood richer, and the child, previously pale and without appetite, improves in its whole constitution. If the enlarged tonsil is very soft, some improvement can be expected from iodine in gargles, and from frequent paintings with the same, long continued. Incisions and scarification are of only temporary value for fresh inflammation or for the evacuation of pus and the inspissated secretion.

Fahnstock's tonsillotome works admirably for children with whom it is impossible to use a knife. The larger tonsil should always be first removed, as often after one operation both will and obedience are lost for a time, but, of course, it is better, where possible, to remove both at one sitting. Ice in large quantities is an excellent styptic and antiphlogistic. I have never yet seen a disagreeably profuse bleeding in children. My young colleagues need not be frightened if a sloughy or diphtheritic deposit is visible on the cut surfaces for a few days, and need not take special measures against it.—*Anton Von Troeltsch, M.D., in Diseases of the Ear in Children.*

---

Cotton Seed Oil in Pharmacy.—Having had occasion during the last six years to manufacture lead plaster in considerable quantities, it occured to me that cotton seed oil might be used instead of olive oil, at less expense, and with as good results. The making of this plaster with cotton seed oil has been questioned, as according to some authorities the product is not of good consistence, and is apt to be soft, sticky, and dark colored, but in my experience such is not the case. If the U. S. P. process is followed in making this plaster, substituting for the olive oil cotton seed oil, and instead of one-half pint of boiling water one and one-half pint are added, the product obtained will be equally as good as that from olive oil. My results with this oil in making lead plaster led me to try it in making the different liniments of the Pharmacopœia, with the following results:

*Linimentum Ammoniæ*—This liniment, made with cotton seed oil, is of much better consistency than when made with olive oil. It is not so thick, will pour easily out of the bottle, and if the ammonia used is of proper strength will make a perfect liniment.

*Linimentum Calcis.*—Cotton seed oil is not at all adapted to making this liniment. It does not readily saponify, separates quickly, and it is almost impossible to unite when separated.

*Linimentum Camphoræ*.—Cotton seed oil is far superior to olive oil in making this liniment, it being a much better solvent of camphor. It has not that disagreeable odor so commonly found in the liniment.

*Linimentum Chloroformi.*—Cotton seed oil being very soluble in chloroform, the liniment made with it leaves nothing to be desired.

*Linimentum Plumbi Subacetatis.*—When liq. plumbi sub-acet. is mixed with cotton seed oil and allowed to stand for some time the oil assumes a reddish color similar to that of freshly-made tincture of myrrh. When the liquor is mixed with olive oil, if the oil be pure no such change takes place. Noticing this change, it occurred to me that this would be a simple and easy way to detect cotton seed oil when mixed with olive oil. This change usually takes place after standing from twelve to twenty-four hours. It is easily detected in mixtures containing five per cent. or even less of the oil, and I am convinced, after making numerous experiments with different oils, that it is peculiar to cotton seed oil.—*S. S. Bradford, Ph. G., in American Jour. of Pharmacy.*

---

Treatment of Iritis.—The first object in the treatment of iritis is to obtain the widest possible dilatation of the pupil and maintain it. It is above all things important to prevent adhesions between the iris and capsule, and this is much more likely to occur when the pupil remains contracted. In the first place, when the pupil is dilated, the iris is removed further from the capsule, making adhesion more unlikely; and, in the second place, the area to be filled with inflammatory exudation is largely increased. and the danger of occlusion of the pupil is correspondingly reduced. This dilatation of the pupil is best obtained by distilling into the eye every six hours a solution of sulphate of atropia of the strength of four grains to the ounce of water. The atropia, being an anodyne, also acts as a calmative to the pain. It acts, too, directly on the inflammatory process, for in dilatation of the pupil the iris tissue is compressed, and of course holds less blood, and as a consequence the exudative process is in a greater or less degree checked. All other treatment is secondary to this. When we see cases of iritis for the first time, the inflammation has usually made such headway that it is useless to attempt to cut it short; but when the pain is excessive and the inflammation of a distinctly sthenic type, local blood-letting is advisable. From three to six leeches to the temple, or the artificial leech will often give immediate relief to these symptoms. But for relief of pain the most reliable remedy is opium in some of its forms. It is often advisable to give the patient an opiate on going to bed—usually as late as ten or eleven o'clock, since the severest pain is commonly felt in the early morning. The opium not only subdues the pain, but acts beneficially on the course of the inflammation. After a full dose of the drug, the injection and photophobia are usually much less. As regards local applications for the relief of pain, nothing is better than warmth and moisture in some form. The most agreeable and convenient manner of its application is by means of a flannel bag about the size of the hand, filled with hops, which is immersed in water as hot as can be borne, and laid on the eye after it has been squeezed so as to expel all superfluous water. It was formerly the custom as soon as a diagnosis of iritis was made out, to begin the use of mercury, and push it rapidly to salivation. The pendulum then swung in the other direction, and there were those who found they could treat iritis, even of the specific form, without any mercury. The proper course lies between these two extremes. In rheumatic iritis mercury seems to exert but little influence on the course of the inflammation, but large doses of salicylate of sodium quite often make a profound impression on it, and in some cases may be expected to cut it short. But it must be given very heroically—as much as thirty grains every three hours until there are giddiness and singing in the ears. Where, however, there is reason to suspect syphilis, even though no positive proof of its existence may be furnished, mercury should be given. It need not be used, however, to the extent of immediate salivation. I am among those who believe that the action of mercury in syphilis is to assist nature in disposing of the excess of cellular elements to which the specific poison has led. This seems to be accomplished by a breaking down or fatty degeneration of the cells, with elimination by the various emunctories. In this process mercury undoubtedly aids, and I think the more effectually when given in moderate doses for a considerable time. Among our out-patients we generally use one-thirty-second to one sixteenth of a grain of bichloride of mercury with two or three grains of iodide of potassium three times daily, after meals.

There is usually no necessity for confining the patient to a dark room. A modulation of the light is all that is requisite. Completely darkened chambers in eye inflammations are among the barbarities which have come down to us from the dark ages of ophthalmology. If all light is positively painful, then the bandage may be applied to exclude it, but we have no right to deprive the other portions of the body and the patient's attendants of the beneficent influence of that stimulant of nature so necessary for the mental and bodily well-being.

In the treatment of the serous form, where there is no pain and but little inflammation, the atropia, opium and other palliative measures may be dispensed with. Here our chief reliance must be on mercury, continued in small doses, for a long time, as the course of the disease is usually very tedious, sometimes running over many months.—*S. M. Burnett, M.D., in Medical News.*

---

Modern Therapeutics.—At the end of a recently published surgical paper occurs this sentence.

"I submit these cases for what they are worth, feeling that any plan of treatment which claims to help us out of the desperate straits in which septic accidents place us, is deserving of a hearing."

The paper gives the details of five cases, one of which terminated fatally; and in two others, at least, the author of the paper says: "The relation in these latter cases, between the use of the acid and the fall of temperature, is not as evident as it might be." Three other cases are mentioned in these words: "With the exception of one case of puerperal septicæmia, one of erythematous erysipelas. and one of lymphangitis, all were successful in result." Of these eight cases, four were successful, and at least two were doubtful, although the author's way of expressing this is, that with these exceptions—six out of eight—"all were successful in result."

The treatment used was a somewhat recently imported novelty, or rather a new and very superficial plausibility, based upon old and doubtful ground. Many, if not most, of the statements made in regard to this novelty are in themselves as irrational and as absurd as any of either domestic or foreign origin since the time of Broussais.

The above mentioned paper was read at a meeting of prominent surgeons, and it and the treatment were discussed, and on the whole the treatment received no support that would be very satisfactory to the author of it. But the remarkable feature of the discussion was, that so far as the published account of it goes, no allusion whatever was made to the irrationality, absurdity and inconsistency of the grounds and statements upon which the treatment was based and was tried. Tacitly, at least, and by fair inference, it seemed to be admitted that there is little or no principle underlying treatment of pathological conditions hitherto difficult to manage, but that "any plan * * * which claims to help us * * * is deserving of a hearing."

Throughout the paper and the discussion it is easy to see that this particular absurdity was accepted with distrust, and "damned with faint praise;" but the wonder still is that a high degree of professional intelligence and reason should ever have permitted it to be accepted at all, and this leads directly to the moral involved in all such medical history.

In the light of such action as this, which is constantly repeated, the medical profession cannot justly condemn the less intelligent laity for empirically trying all the quackeries that are proposed to them. Such an irrational "plan of treatment" as this, presented in such a way, is to the profession an almost exact analogue to "Pond's Extract" to the laity. Both claim to be effective for good, and although there is but little reason or intelligent support for either, still let them be tried lest something useful should escape by being irrational.

Whether it be a variety of turpentine that only one man knows, or a carbolic acid that only one man makes, or water from witch hazel twigs that only one man can distill, the principle or want of rational principle is the same, and the inconsistencies are the same. If the profession weakly lends itself to try every plausible novelty that may be presented to it without first inquiring closely whether its pretentions be rational or not, or be supported by the known relations between cause and effect, then it cannot justly blame the people for persistently trying one so-called system of medicine after another, and one patent medicine after another throughout the long list of hurtful, popular errors. Neither can the profession hope to make progress in its battle for life against such error from so weak a position.

At a Grecian council of war, upon the demoralization of the Greek leaders, held after Troy had been unsuccessfully besieged for seven years, Ulysses is represented to have said to Agamemnon—

> " And 'tis this fever that keeps Troy on foot,
> Not her own sinews. To end a tale of length,
> Troy in our weakness stands, not in her strength "

—*Squibbs Ephemeris of Materia Medica, etc.*

---

Medical Diploma Scandal.—The original medical colleges, which have been organized to supply a real want, alone have a right to existence. After the needs of the country have been satisfied, colleges go on organizing, not in obedience to any legitimate requirement, but merely to satisfy the ambition of professorial aspirants.

There is, however, usually in these smaller cities one leading doctor who organizes a faculty from the material of the town, or of the neighborhood, an old warehouse is converted into a college building, and clinics chiefly surgical, and made up of the patients of the surgeon, are commenced, with the intention to advertise the great man as their real object. When one medical school is organized, a second must follow. A faculty created from the adherents of one or two great men, does not provide for the remaining "medical talent" of the town, and hence great dissatisfaction is felt. Very soon a new organization is arranged, and the "just claims to recognition" of the hitherto unrecognized professors are suitably acknowledged. In some instances the city or town having but two or three sufficiently eminent men for the practical chairs, an anatomist, or physiologist, or professor of some other scientific department, is summoned from some distant city. Then the pretence for organizing a new college, is the recognition of "home talent." What motive soever may be alleged for founding the college, the real intention is to obtain the title of Professor, and the supposed privileges and benefits thereunto belonging. Unfortunately the title becomes cheap, sometimes actually goes a begging in the smaller cities with three or more colleges, for presently the distinction consists in not being a *professor*. Indeed we know of one city, where it is usual in the eulogistic funeral discourses, to say of the eminent deceased, he had been frequently importuned to accept this or that chair in the medical colleges, but had invariably declined.—*Medical News.*

---

THE MOOR BATH.—These moor baths are curious affairs. Here in Austria they excavate a marsh, where decaying vegetable mould and water is in abundance, cart it to the bath houses, grind it in a mill and pass it into a large tank where it is mixed with water and heated by steam. From the tank it is drawn off into portable bath tubs, where it is again mixed, more water added and the temperature brought to the desired grade. Then it is wheeled into the bath room and the unfortunate bather plunges into a black, bad smelling mixture where he remains from twenty minutes to half an hour, when he steps into a second tub full of pure water to cleanse himself. The moor is too precious to be wasted, so after being used it is deposited in a great heap, where it is said to remain ten years before it will be fit for use a second time. These heaps remind one in more ways than one of those which the dairy farmer accumulates behind his cow stable. The moor bath is popularly believed to be very beneficial in all cases—a veritable cure-all. The physicians questioned on the subject were unwilling to commit themselves as to its indications and effects, except in the case of hypochondriacs, with one exception, and he said where a poultice for the whole body was necessary he knew of no more convenient method of applying it. It contains the products of the decomposition of vegetable substances and a considerable quantity of iron salts and glauber's salts, but the chief requisites seem to be that the bath when prepared should smell as badly as possible and cost a good round sum.—*H. L. T., in Cin. Lancet and Clinic.*

---

Alcoholic Anæsthesia.—We recognize two forms of Alcoholic Anæsthesia :—

*First.* A condition of ancesthesia following the administration of a certain quantity of alcohol. The state of the person borders upon and may lapse finally into alcoholic coma, although it is not necessary that the patient should be profoundly under alcoholic influence to manifest an anæsthetic condition. This form of anæsthesia is general in character and seems to affect not only the integumentary but the deeper tissues of the body as well. It is transitory in its effects and passes off as the alcohol is eliminated from the system, the patient recovering his normal sensory condition when this occurs.

*Second.* A condition of anæsthesia due to the long continued action of alcohol on the nervous centers. This form is partial in character affecting a limited space; it is, however, of comparatively long duration, lasting some weeks or months, the patient slowly recovering his normal sensibility after a period of abstinence and the use of proper therapeutic measures.

To illustrate the anæsthetic value of alcohol we have only to go back to the old days of surgery when ether and chloroform were unknown, when the usual custom was to prepare the patient for a painful and protracted operation by partially intoxicating him with alcohol, in order to reduce the amount of physical suffering to a minimum.

In certain forms of neuralgia the value of a few glasses of wine or a tumbler of brandy and water has been recognized by the medical authorities. In the case of invalids, when the extraction of teeth was necessary, and when, for good reasons, the usual anæsthetics were not regarded as safe, it has been my custom to advise the use of alcohol as a substitute with the desired effect.—*L. D. Mason, M D., in Journal of Inebriety.*

---

SPECIALTIES.—The existence of specialties was a logical sequence of an accumulated store of knowledge, and, while it cannot be denied that their exercise is liable to much abuse, and may, in the hands of the unworthy, prove a stepping-stone to deceitful and fraudulent practices, the same remark might be applied with equal justice to any of the mechanical arts. We must, in all our dealings, place a certain amount of confidence in the honesty of human nature until experience shall have taught the contrary, and we have no more means of judging of the skill of a watchmaker, to whom an instrument is brought for repairs, than of the particular fitness of a man who represents himself as specially qualified to treat a certain class of cases. It is presumed in both instances that the necessary amount of study and careful training has preceded the assumption of any responsibility, and it is to be remarked, to the credit of the race, that, in the majority of instances, the presumption is well founded. Of the way in which specialties grow, after once having taken root, no better idea could be obtained than the following extract, taken from the writings of one of the most eminent of English authors of the present day, Mr Jonathan Hutchinson: "In the early stage of any department of knowledge it is almost a matter of necessity that it should be in the hands of a few; but it is the highest privilege of those who thus devote themselves to the reclaiming of new spots of territory, to be able after a while to hand them over to the commonwealth, to prove that they are now cultivated and well worthy of annexation.—*Joseph Eichberg, M.D., in Cin. Lancet and Clinic.*

---

Contagiousness of Pulmonary Consumption.—Dr. Herbert Davies, consulting physician to the London Hospital, and to the Royal Hospital for Diseases of the Chest, writes to a recent number of the *British Medical Journal*, giving some strong arguments against the contagious properties of pulmonary consumption. He quotes from a letter, written in 1867, by Mr. Edwards (resident medical officer of the Brompton Hospital for seventeen years), to Dr. R. Payne Cotton, in which he says that he remembers fifty nine resident medical assistants, whose duration of office has averaged six months, all but two of whom are living, one dying from aneurism, and the other from some unknown cause. The present chaplain has held office for seventeen years, and his two predecessors are living. The matron has been resident for more than sixteen years, and the two former matrons are living. Of the nurses now in residence, one has been here 24 years; two, 12 years; one, 8 years; one 7, one 6½, and one 5 years. No under-nurse has died of phthisis. The head nurses sleep each in a room containing fifty patients, and two only are known to have died—one from apoplexy; and one, some time after she had left the hospital, and after an unhappy married life, of phthisis. All but two of the physicians who have attended the in and out patients during years are living. One died from causes unknown, the other from causes unconnected with diseases of the lung.—*Med. and Surg. Reporter.*

---

Cure of Glycosuria and Diabetes Mellitus by Bromide of Potassium.—Dr. Felizet, having had occasion to administer bromide of potassium to a diabetic patient subject to nervous disorders, noticed that the quantity of sugar diminished in proportion as the nervous troubles became better; he then made a series of experiments to determine in how far the bromide exerted any influence. Having rendered several animals diabetic by pricking the fourth ventricle and immediately giving intravenous injections of the remedy, he soon observed a diminution of sugar in the urine. Fifteen cases carefully observed during six years, permit him to affirm the cure of glycosuria and diabetes mellitus by the bromide of potassium; he supplements treatment by other means calculated to render useful aid.—*Bulletin de l'Acad. de Med.—St. Louis Med. & Surg. Journal.*

---

THE SEA-SIDE SANITARY HOTEL OF THE FUTURE.—Anxious guest to hall-boy: "Boy, where are the water closets?" "Hain't got any, sir; they breeds fever. Boat goes down the harbor every morning. Ladies at nine, gentlemen at ten." "Well, is dinner ready?" "No, sir. We always carbolize the dining room before meals. Now they are spraying the waiters, sir." Impatiently: "Well, where is your ice water?" "Don't have drinking water now, sir. 'Tain't healthy. Yonder's our Labarraque mixture flavored to taste. Have a glass, sir?" Guest retires and takes a thymolized julep.—*Sanitary Engineer.*

---

MEMBRANOUS DYSMENORRHŒA.—Dr. Lutand (Ann. de Gynecol.) concludes from a study of this subject: 1st. That membraneous dysmenorrhœa consists of an exfoliation of the hypertrophied uterine mucous membrane. 2d. The expulsion of the mucous membrane does not take place each month; several months may elapse between each expulsion. 3d. This affection is quite common in virgins, and can, therefore, not be regarded as the result of conception. 4th. It nearly always causes sterility.—*Chicago Med. Review.*

---

POMADE FOR COMEDONES.—The *St. Louis Medical Journal* says that Una, in *Virchow's Archives*, recommended the following for comedones: Kaolin, four parts; glycerine, three parts; acetic acid, two parts, with or without the addition of a small quantity of some ethereal oil. With this pomade he covers the parts affected, in the evening, and if need be during the day. The comedones can be easily expressed after several days, most of them coming out by washing the parts with pomice-stone soap.—*Chicago Med. Review.*

